# Supplementary figures and images for: A stress-response-related inter-compartmental signalling pathway regulates embryonic cuticle integrity in Arabidopsis
Source: PLoS Genet. 2019 Apr 18;15(4):e1007847. doi: 10.1371/journal.pgen.1007847 (PMC6490923; doi:10.1371/journal.pgen.1007847)

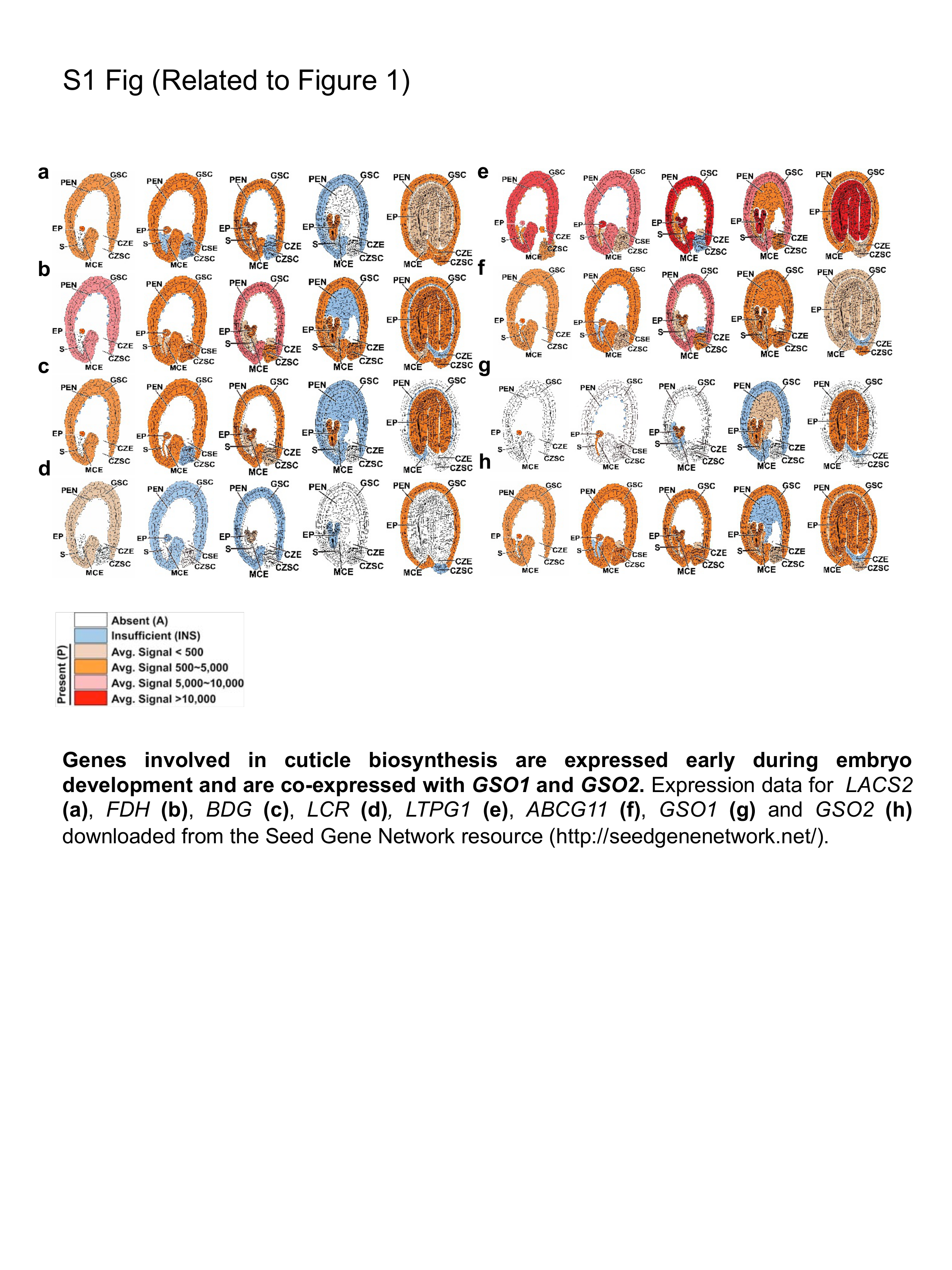

Supplement: S1 Fig — Expression data for LACS2 (a), FDH (b), BDG (c), LCR (d), LTPG1 (e), ABCG11 (f), GSO1 (g) and GSO2 (h) downloaded from the Seed Gene Network resource (http://seedgenenetwork.net/). (TIF) [file pgen.1007847.s001.tif]

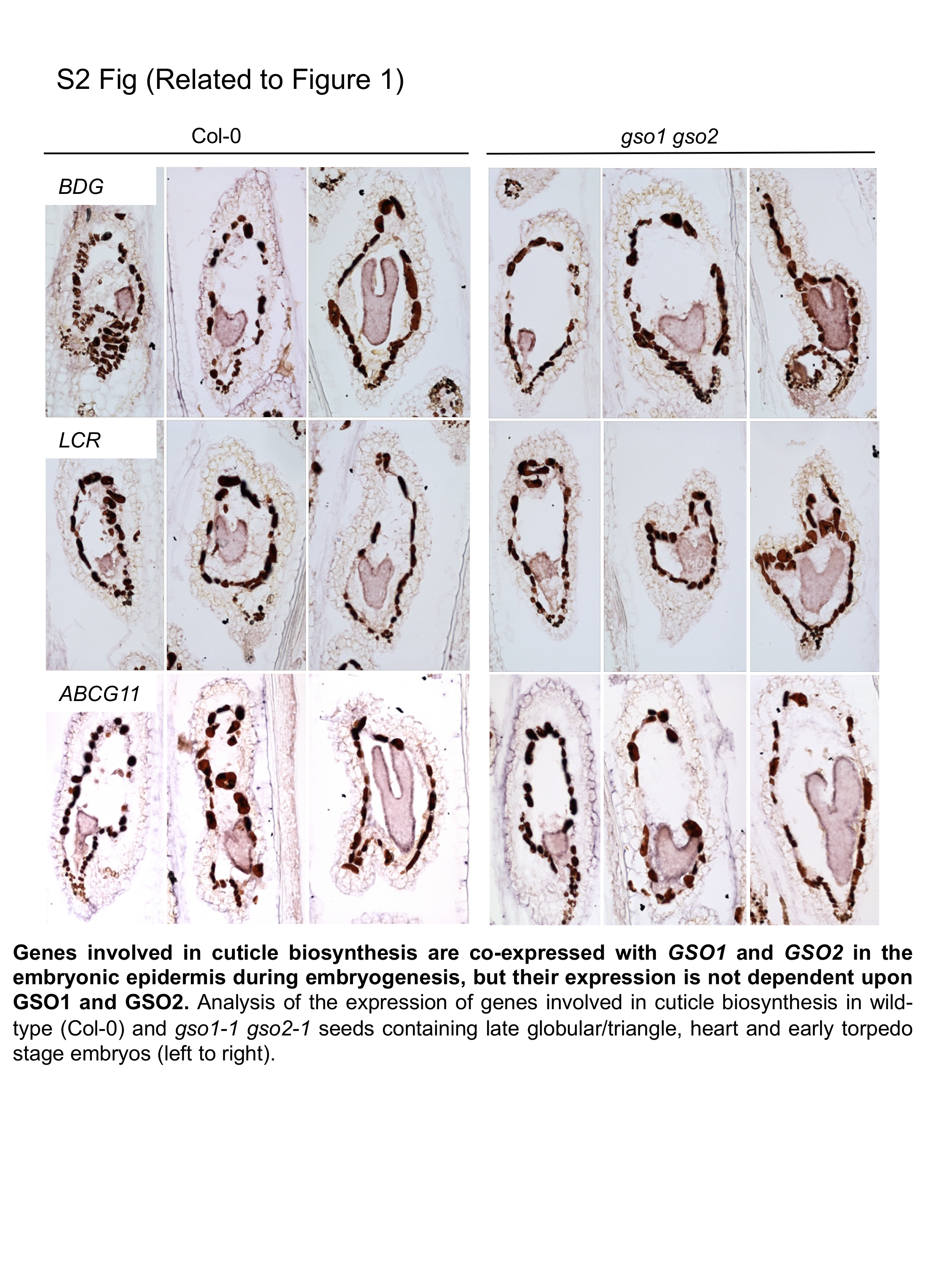

Supplement: S2 Fig — Analysis of the expression of genes involved in cuticle biosynthesis in wild-type (Col-0) and gso1-1 gso2-1 seeds containing late globular/triangle, heart and early torpedo stage embryos (left to right). (TIF) [file pgen.1007847.s002.tif]

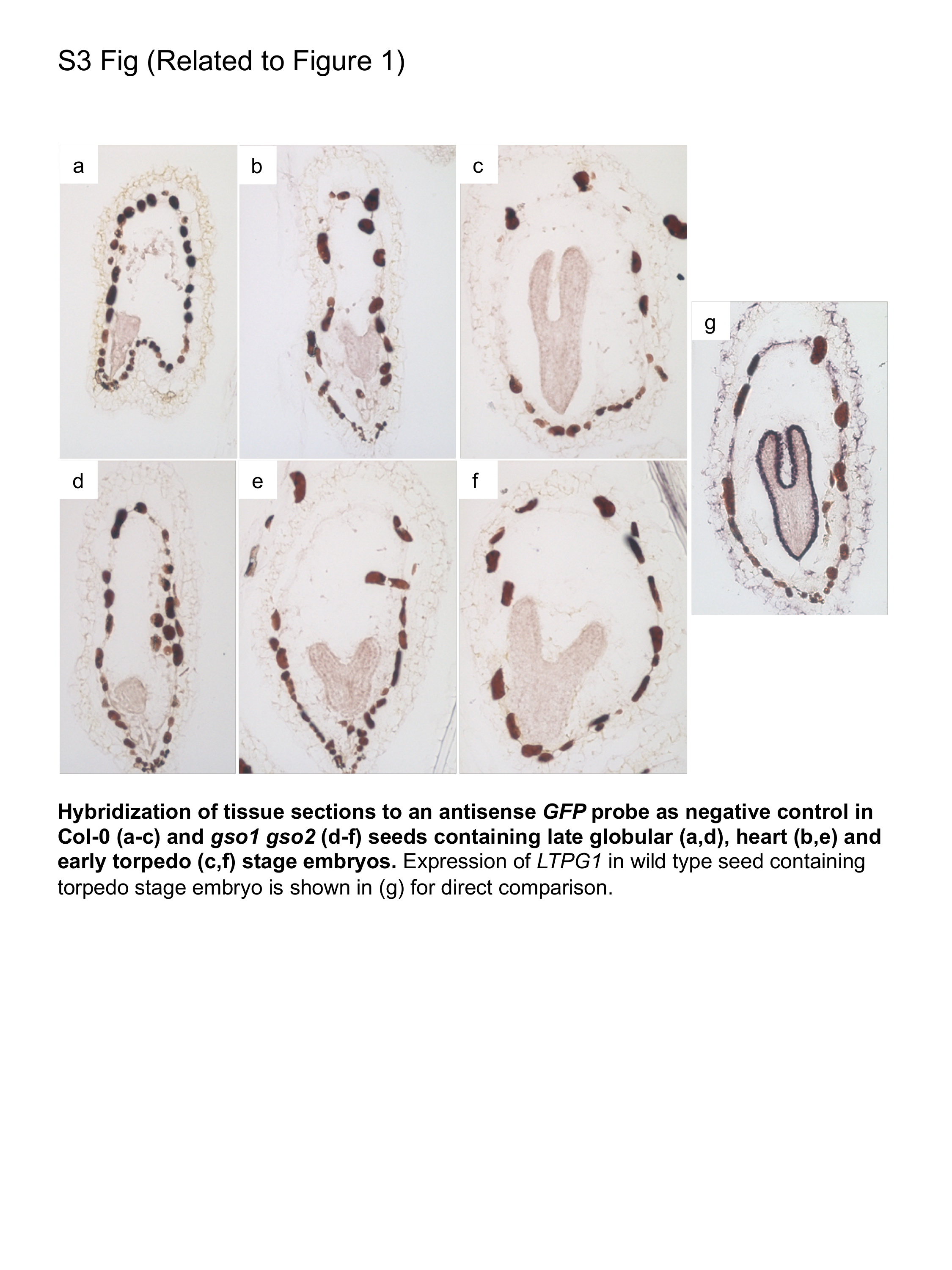

Supplement: S3 Fig — (Related to Fig 1): Hybridization of tissue sections to an antisense GFP probe as negative control in Col-0 (a-c) and gso1 gso2 (d-f) seeds containing late globular (a,d), heart (b,e) and early torpedo (c,f) stage embryos. Expression of LTPG1 in wild type seed containing torpedo stage embryo is shown in (g) for direct comparison. (TIF) [file pgen.1007847.s003.tif]

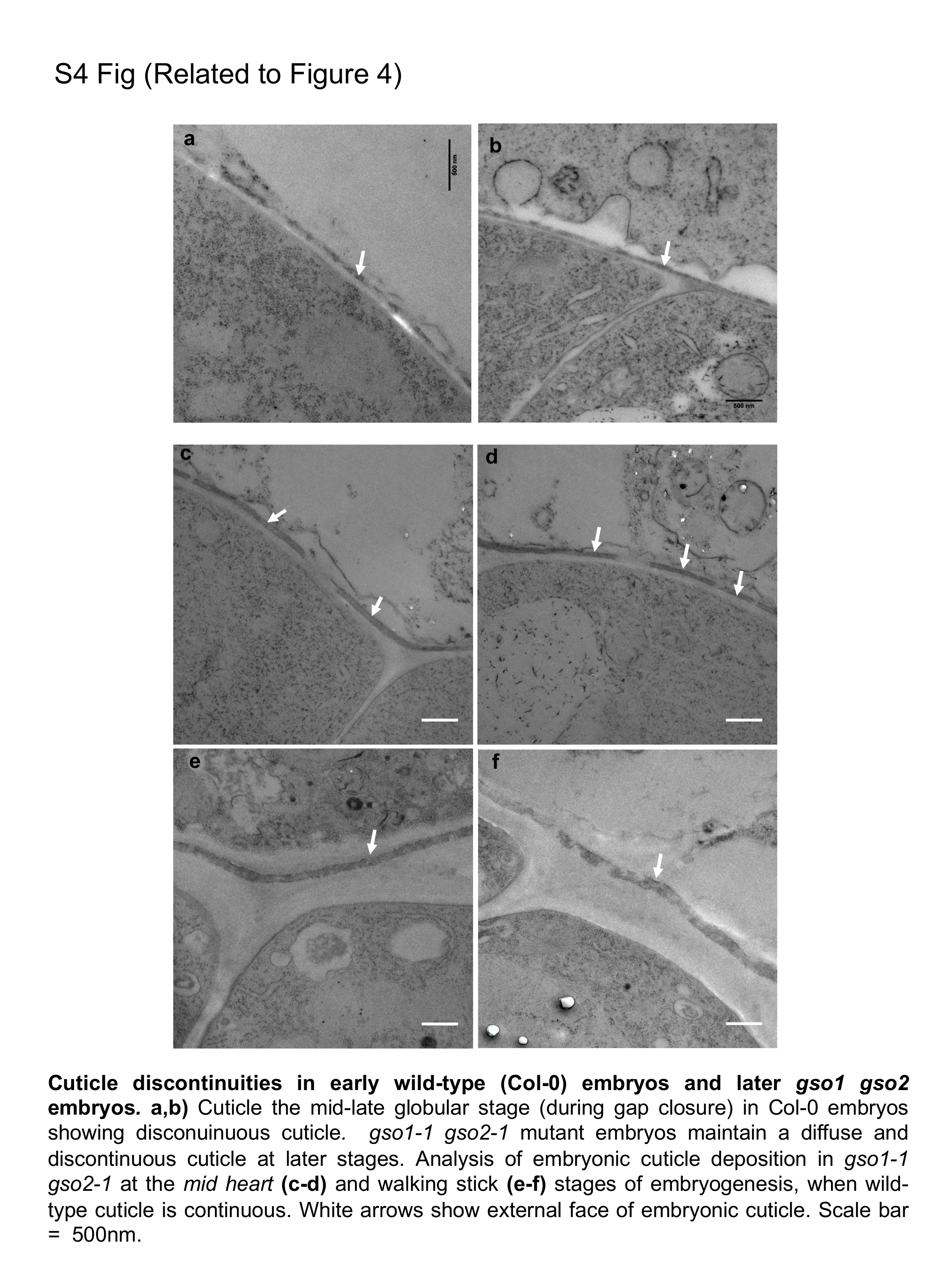

Supplement: S4 Fig — a,b) Cuticle the mid-late globular stage (during gap closure) in Col-0 embryos showing disconuinuous cuticle. gso1-1 gso2-1 mutant embryos maintain a diffuse and discontinuous cuticle at later stages. Analysis of embryonic cuticle deposition in gso1-1 gso2-1 at the mid heart (c-d) and walking stick (e-f) stages of embryogenesis, when wild-type cuticle is continuous. White arrows show external face of embryonic cuticle. Scale bar = 500nm. (TIF) [file pgen.1007847.s004.tif]

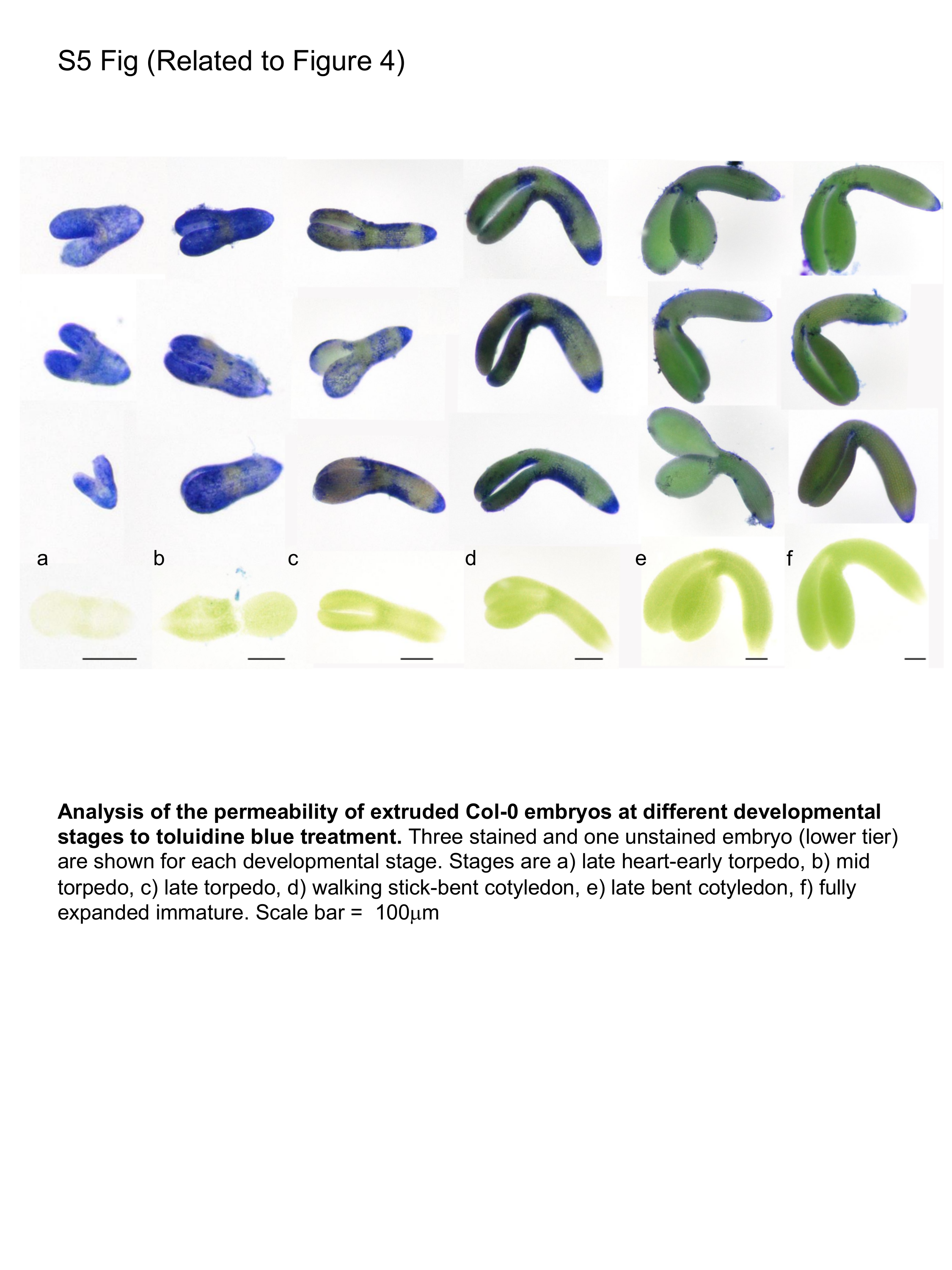

Supplement: S5 Fig — Three stained and one unstained embryo (lower tier) are shown for each developmental stage. Stages are a) late heart-early torpedo, b) mid torpedo, c) late torpedo, d) walking stick-bent cotyledon, e) late bent cotyledon, f) fully expanded immature. Scale bar = 100μm. (TIF) [file pgen.1007847.s005.tif]

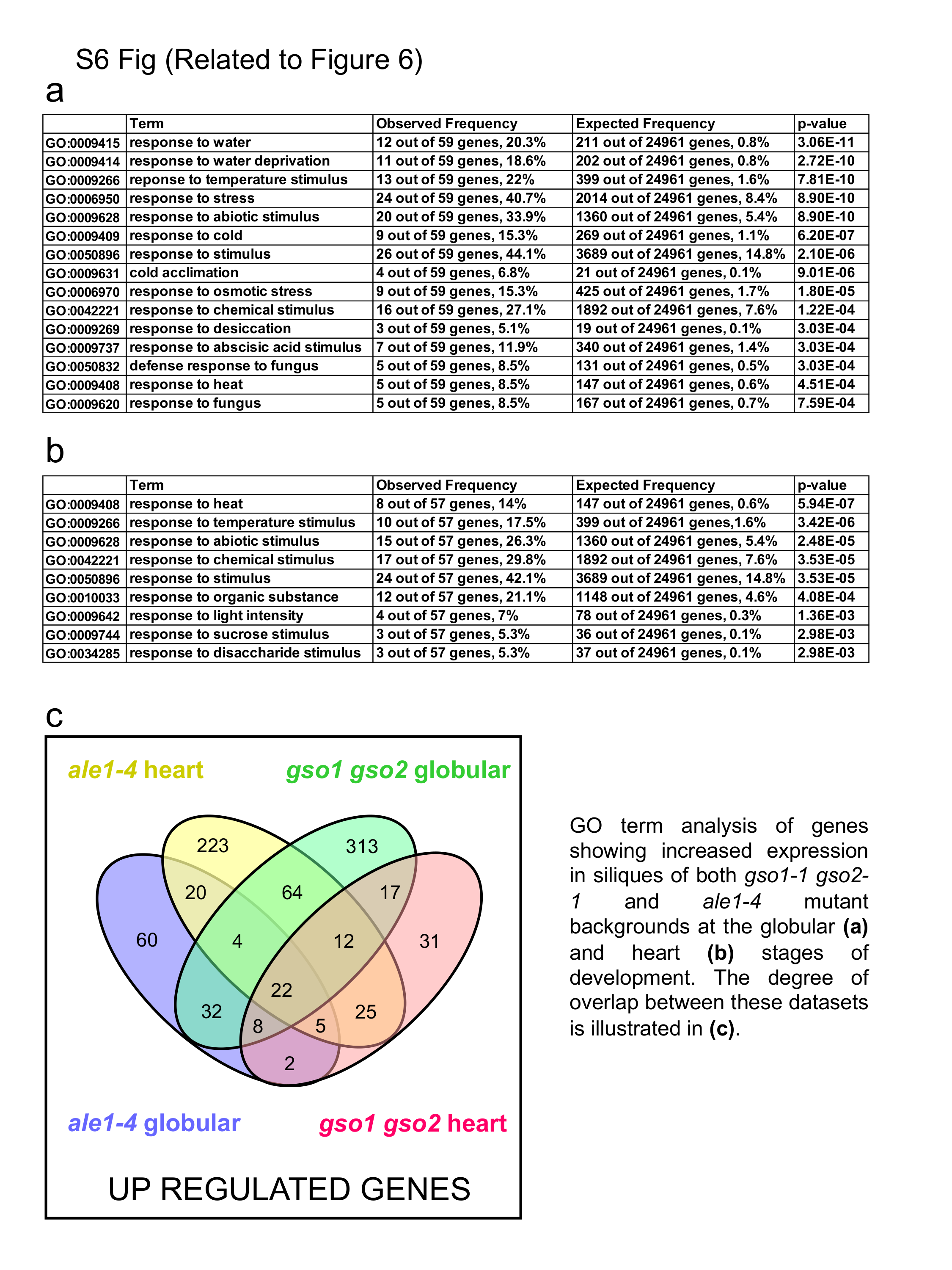

Supplement: S6 Fig — (Related to Fig 6): GO term analysis of genes showing increased expression in siliques of both gso1-1 gso2-1 and ale1-4 mutant backgrounds at the globular (a) and heart (b) stages of embryo development. The degree of overlap between these datasets is illustrated in (c). (TIF) [file pgen.1007847.s006.tif]

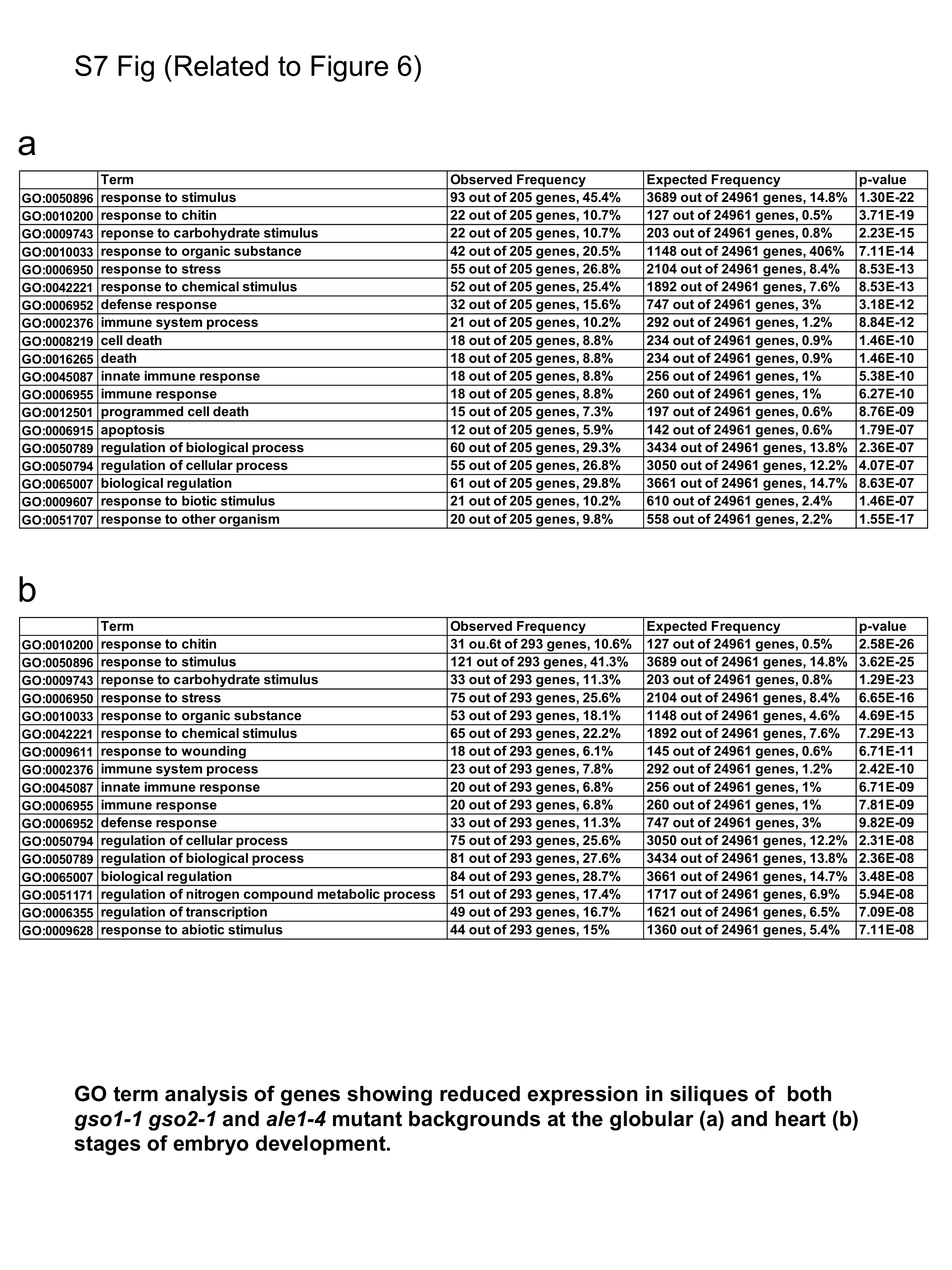

Supplement: S7 Fig — (Related to Fig 6): GO term analysis of genes showing reduced expression in siliques of both gso1-1 gso2-1 and ale1-4 mutant backgrounds at the globular (a) and heart (b) stages of embryo development. (TIF) [file pgen.1007847.s007.tif]

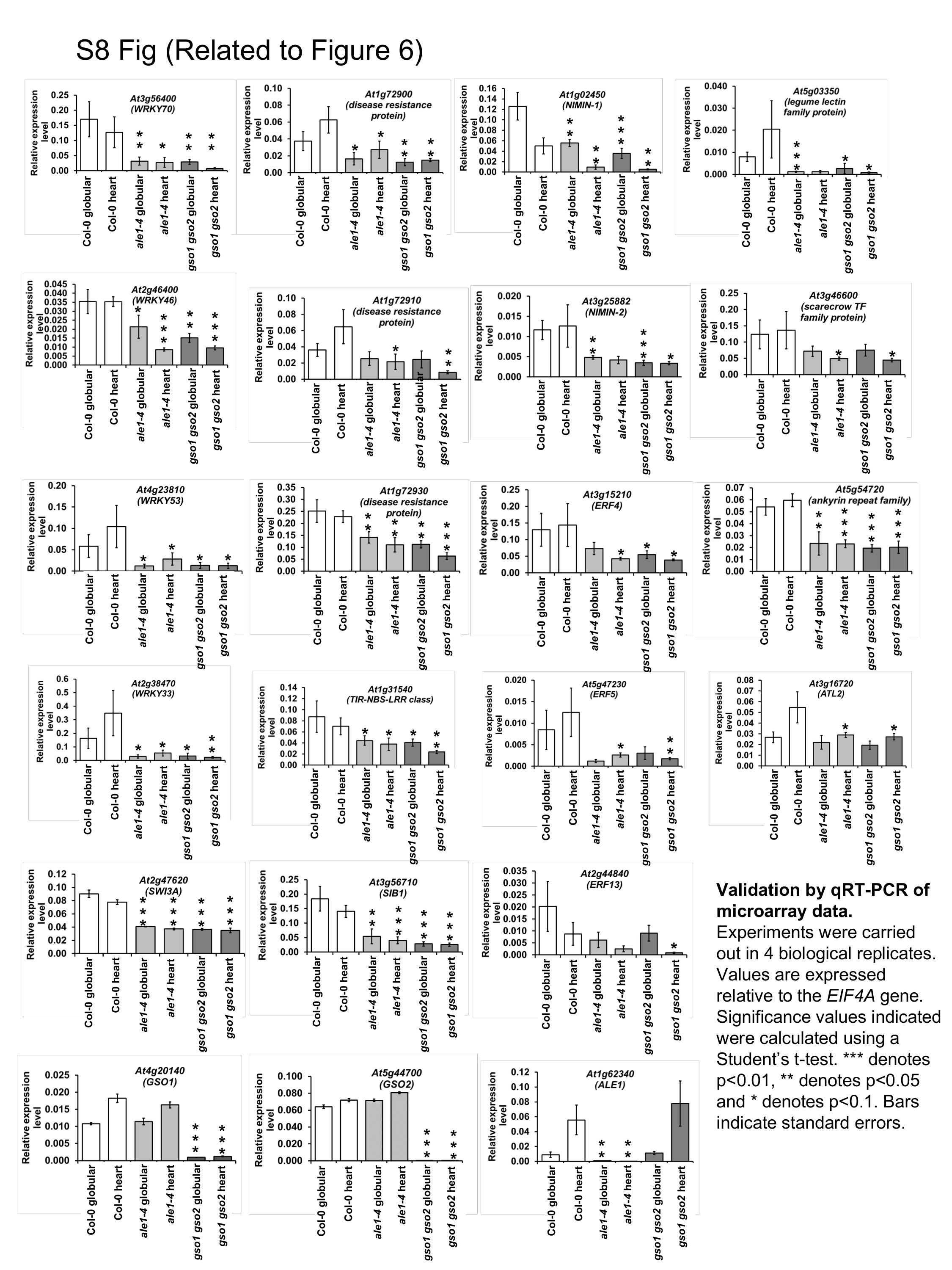

Supplement: S8 Fig — Experiments were carried out in 4 biological replicates. Values are expressed relative to the EIF4A gene. Significance values indicated were calculated using a Student’s t-test. *** denotes p<0.01, ** denotes p<0.05 and * denotes p<0.1. Bars indicate standard errors. (TIF) [file pgen.1007847.s008.tif]

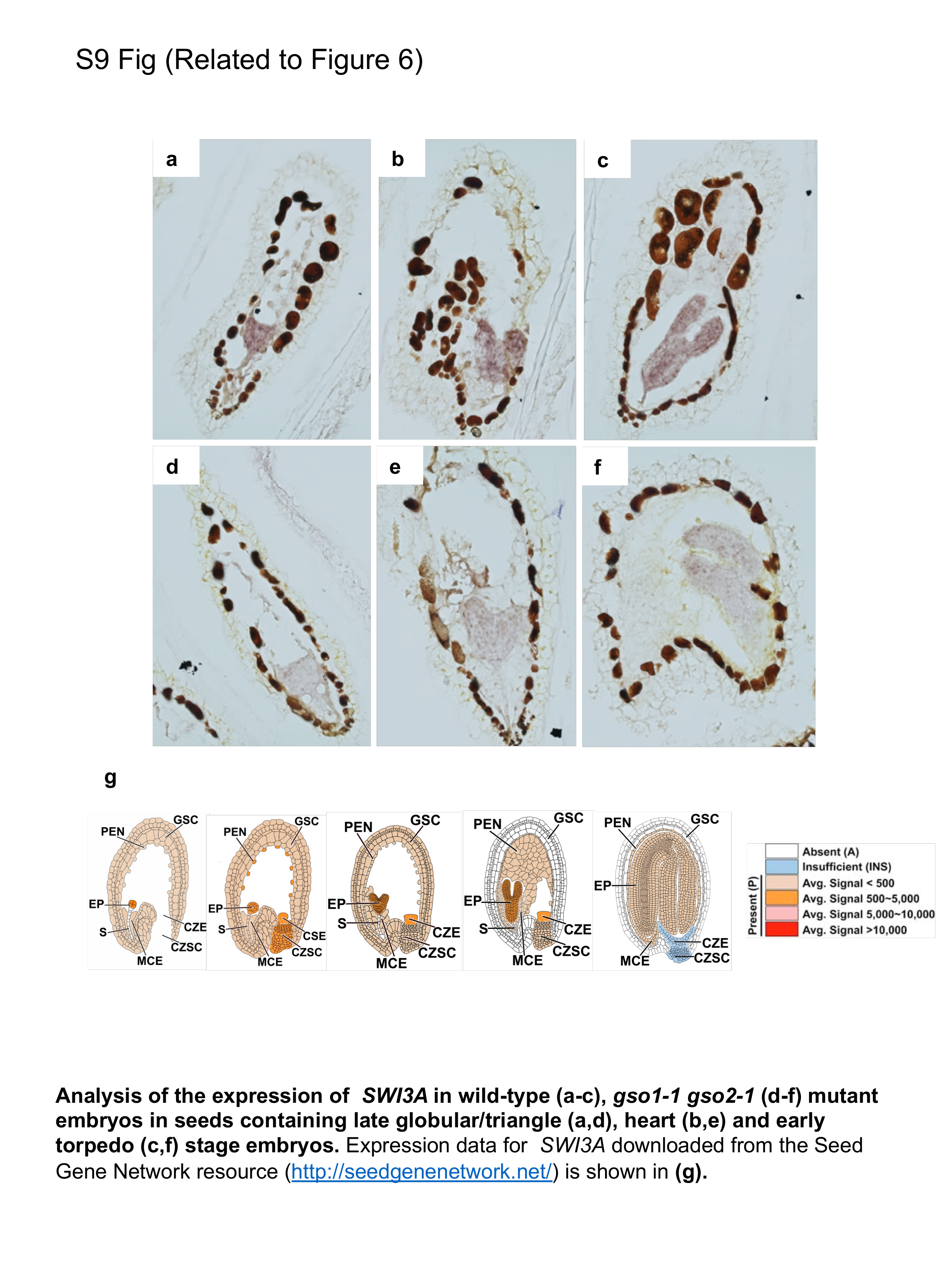

Supplement: S9 Fig — (Related to Fig 6): Analysis of the expression of SWI3A in wild-type (a-c), gso1-1 gso2-1 (d-f) mutant embryos in seeds containing late globular/triangle (a,d), heart (b,e) and early torpedo (c,f) stage embryos. Expression data for SWI3A downloaded from the Seed Gene Network resource (http://seedgenenetwork.net/) is shown in (g). (TIF) [file pgen.1007847.s009.tif]

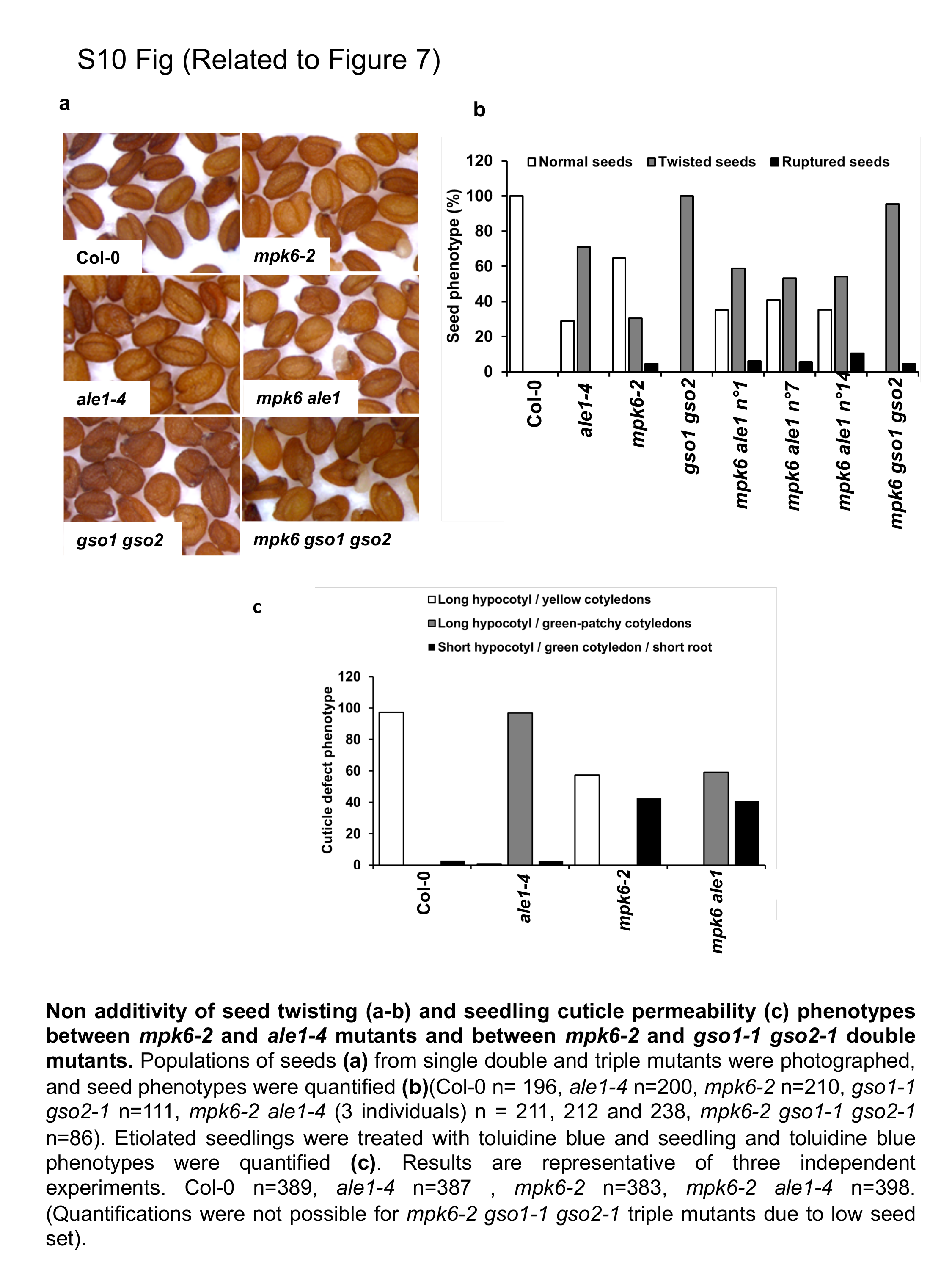

Supplement: S10 Fig — (Related to Fig 7): Non additivity of seed twisting (a-b) and seedling cuticle permeability (c) phenotypes between mpk6-2 and ale1-4 mutants and between mpk6-2 and gso1-1 gso2-1 double mutants. Populations of seeds (a) from single double and triple mutants were photographed, and seed phenotypes were quantified (b)(Col-0 n = 196, ale1-4 n = 200, mpk6-2 n = 210, gso1-1 gso2-1 n = 111, mpk6-2 ale1-4 (3 individuals) n = 211, 212 and 238, mpk6-2 gso1-1 gso2-1 n = 86). Etiolated seedlings were treated with toluidine blue and seedling and toluidine blue phenotypes were quantified (c). Results are representative of three independent experiments. Col-0 n = 389, ale1-4 n = 387, mpk6-2 n = 383, mpk6-2 ale1-4 n = 398. (Quantifications were not possible for mpk6-2 gso1-1 gso2-1 triple mutants due to low seed set). (TIF) [file pgen.1007847.s010.tif]

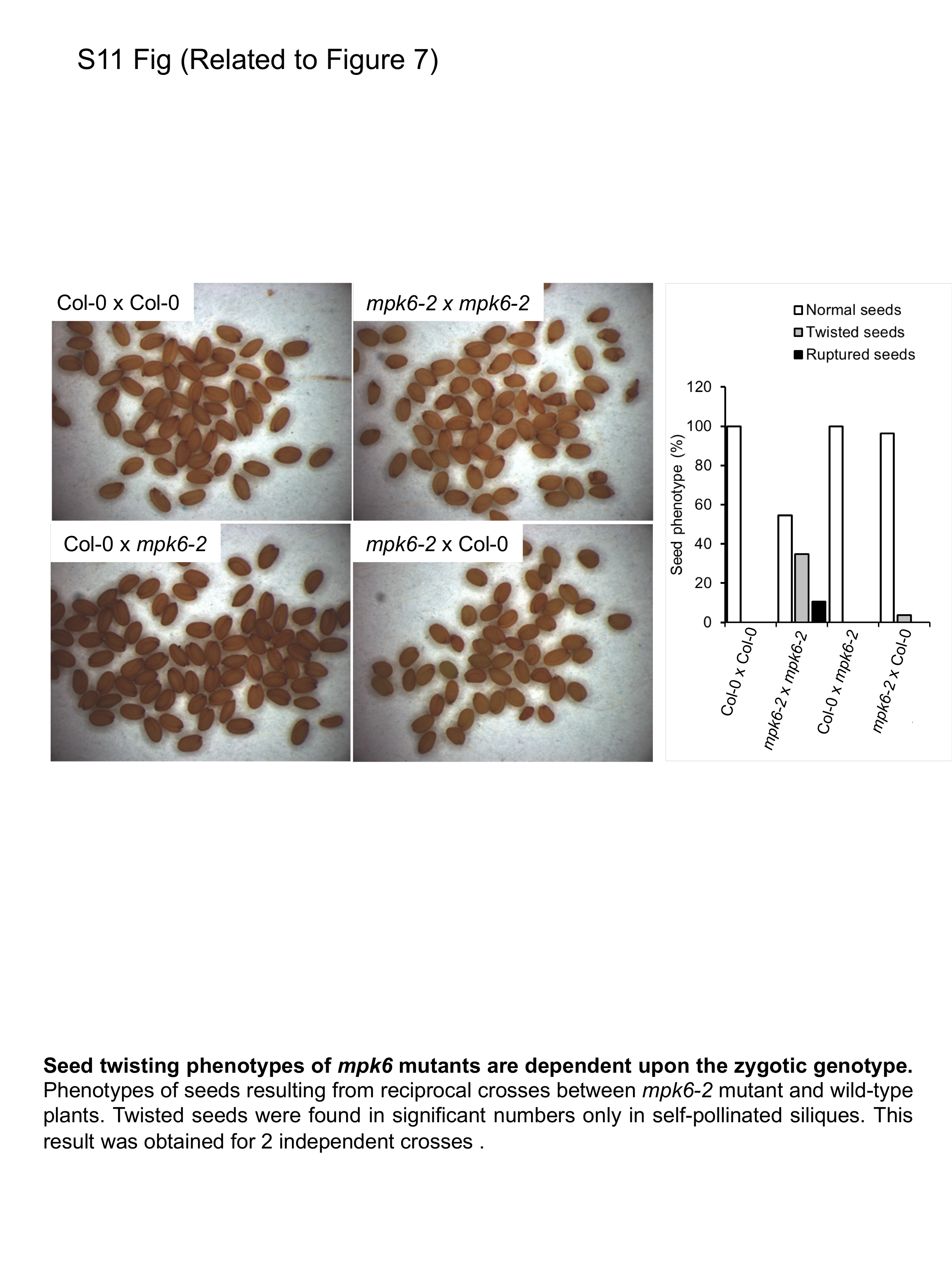

Supplement: S11 Fig — Phenotypes of seeds resulting from reciprocal crosses between mpk6-2 mutant and wild-type plants. Twisted seeds were found in significant numbers only in self-pollinated siliques. This result was obtained for 2 independent crosses. (TIF) [file pgen.1007847.s011.tif]

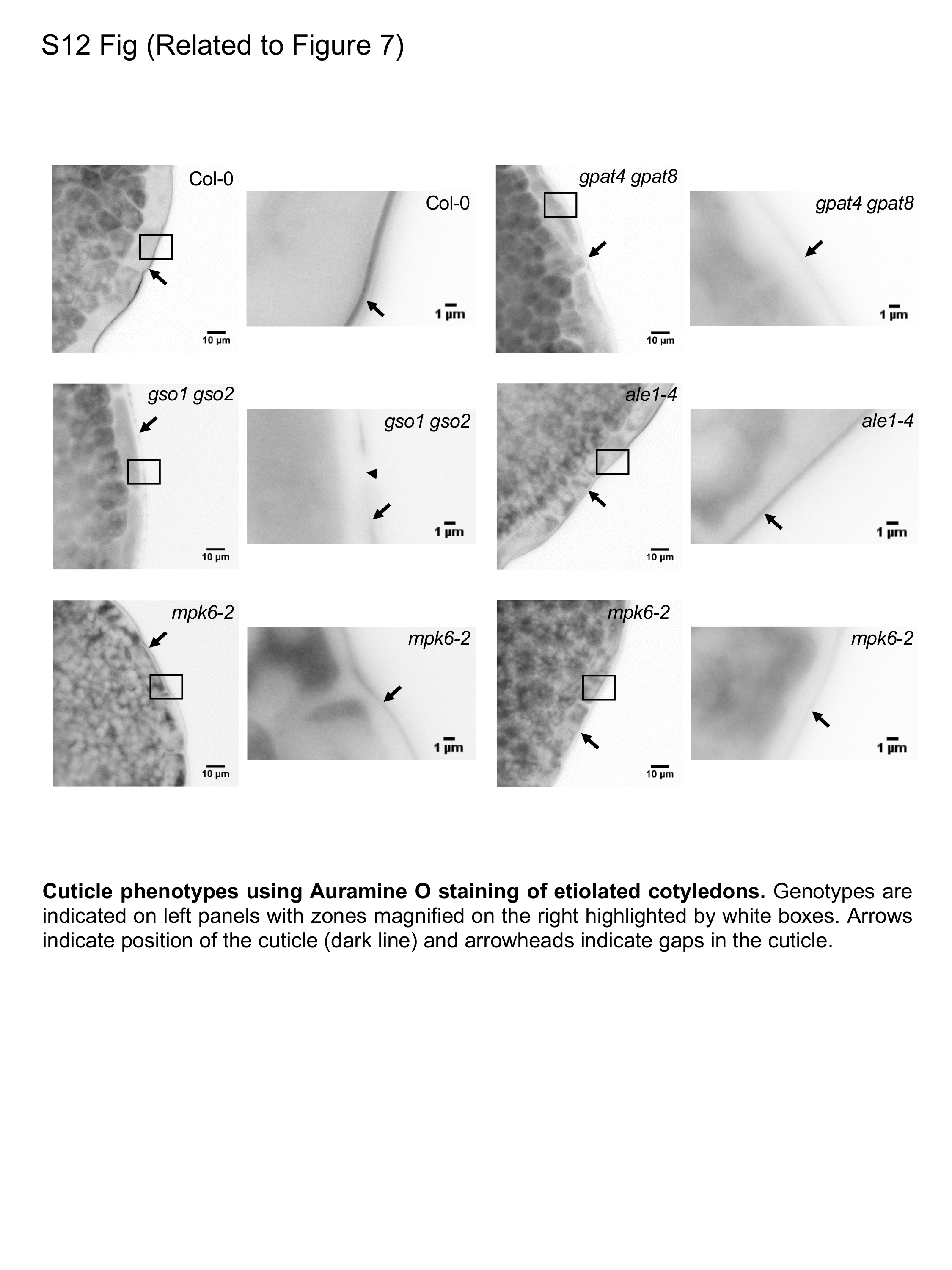

Supplement: S12 Fig — Genotypes are indicated on left panels with zones magnified on the right highlighted by white boxes. Arrows indicate position of the cuticle (dark line) and arrowheads indicate gaps in the cuticle. (TIF) [file pgen.1007847.s012.tif]

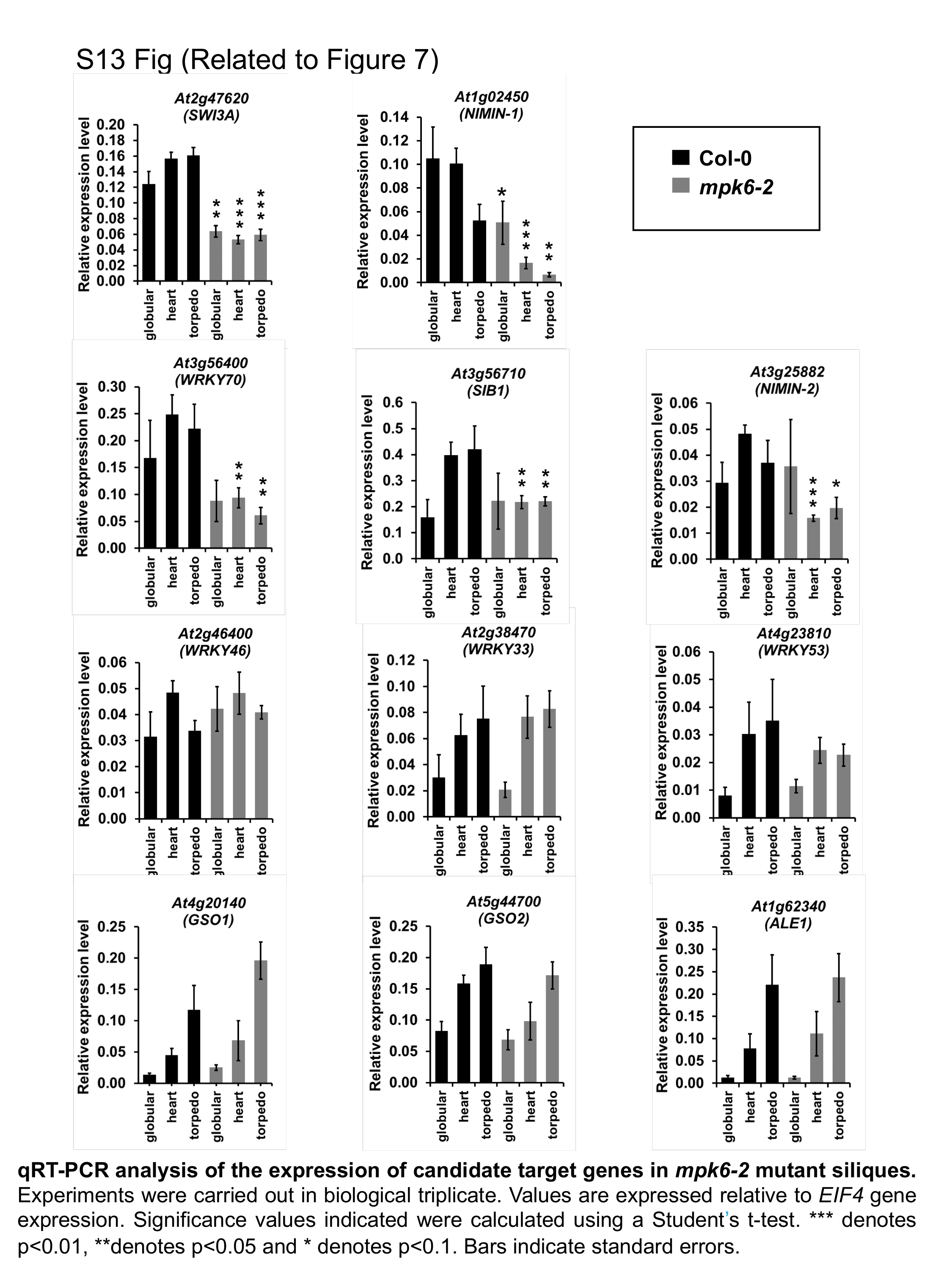

Supplement: S13 Fig — Experiments were carried out in biological triplicate. Values are expressed relative to EIF4 gene expression. Significance values indicated were calculated using a Student’s t-test. *** denotes p<0.01, **denotes p<0.05 and * denotes p<0.1. Bars indicate standard errors. (TIF) [file pgen.1007847.s013.tif]

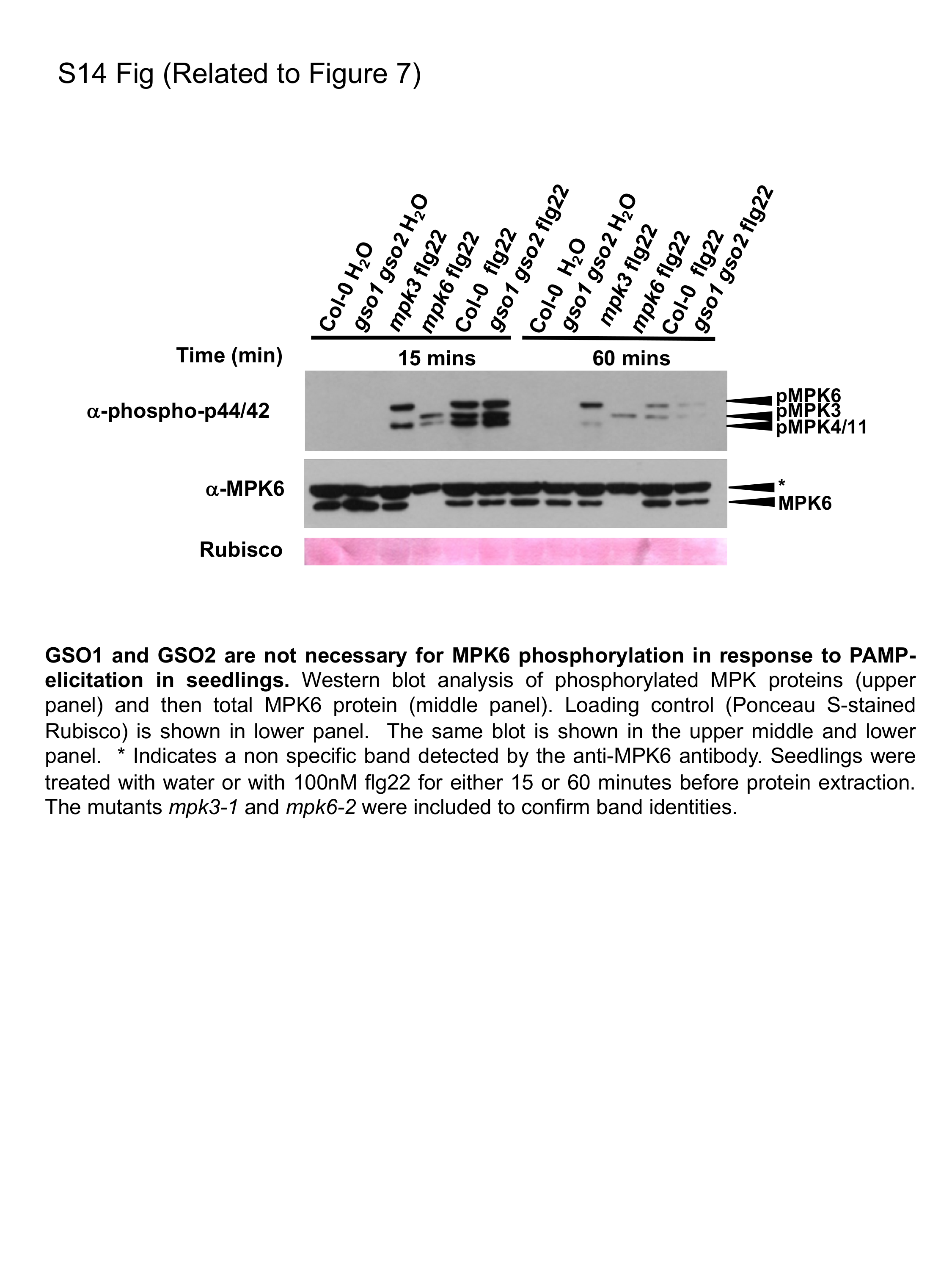

Supplement: S14 Fig — Western blot analysis of phosphorylated MPK proteins (upper panel) and then total MPK6 protein (middle panel). Loading control (Ponceau S-stained Rubisco) is shown in lower panel. The same blot is shown in the upper middle and lower panel. * Indicates a non specific band detected by the anti-MPK6 antibody. Seedlings were treated with water or with 100nM flg22 for either 15 or 60 minutes before protein extraction. The mutants mpk3-1 and mpk6-2 were included to confirm band identities. (TIF) [file pgen.1007847.s014.tif]

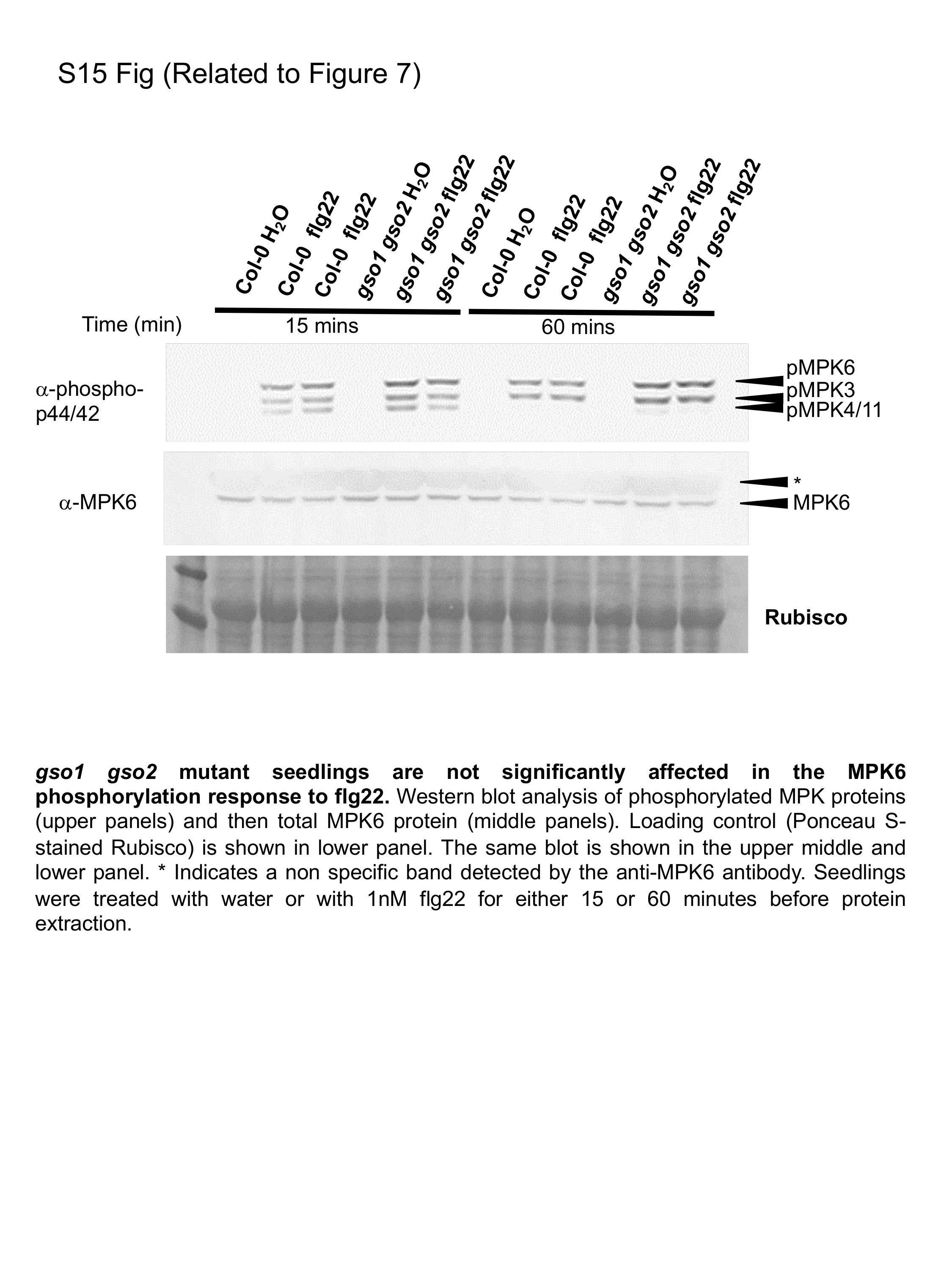

Supplement: S15 Fig — Western blot analysis of phosphorylated MPK proteins (upper panels) and then total MPK6 protein (middle panels). Loading control (Ponceau S-stained Rubisco) is shown in lower panel. The same blot is shown in the upper middle and lower panel. * Indicates a non specific band detected by the anti-MPK6 antibody. Seedlings were treated with water or with 1nM flg22 for either 15 or 60 minutes before protein extraction. (TIF) [file pgen.1007847.s015.tif]

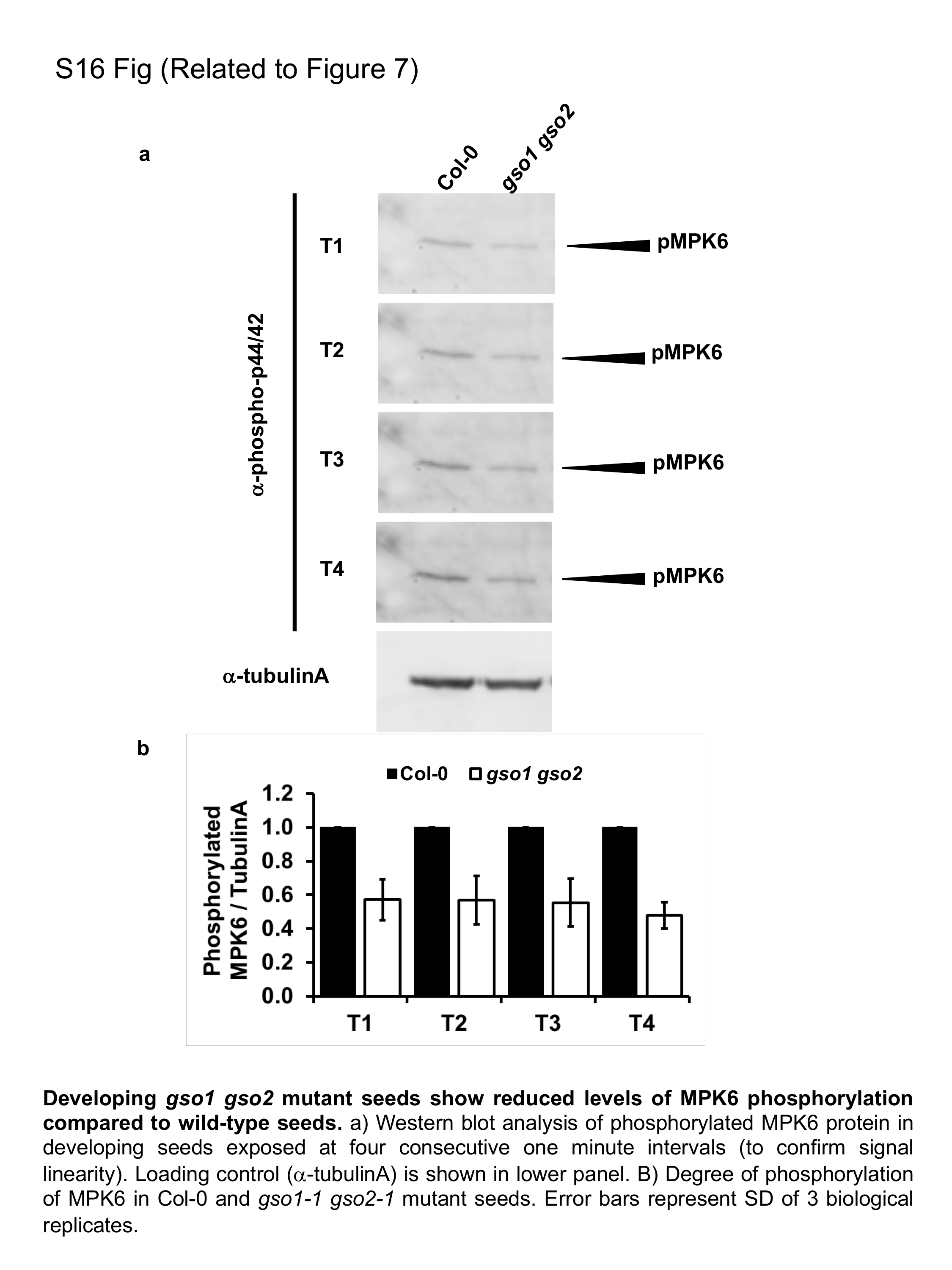

Supplement: S16 Fig — a) Western blot analysis of phosphorylated MPK6 protein in developing seeds exposed at four consecutive one minute intervals (to confirm signal linearity). Loading control (α-tubulinA) is shown in lower panel. B) Degree of phosphorylation of MPK6 in Col-0 and gso1-1 gso2-1 mutant seeds. Error bars represent SD of 3 biological replicates. (TIF) [file pgen.1007847.s016.tif]

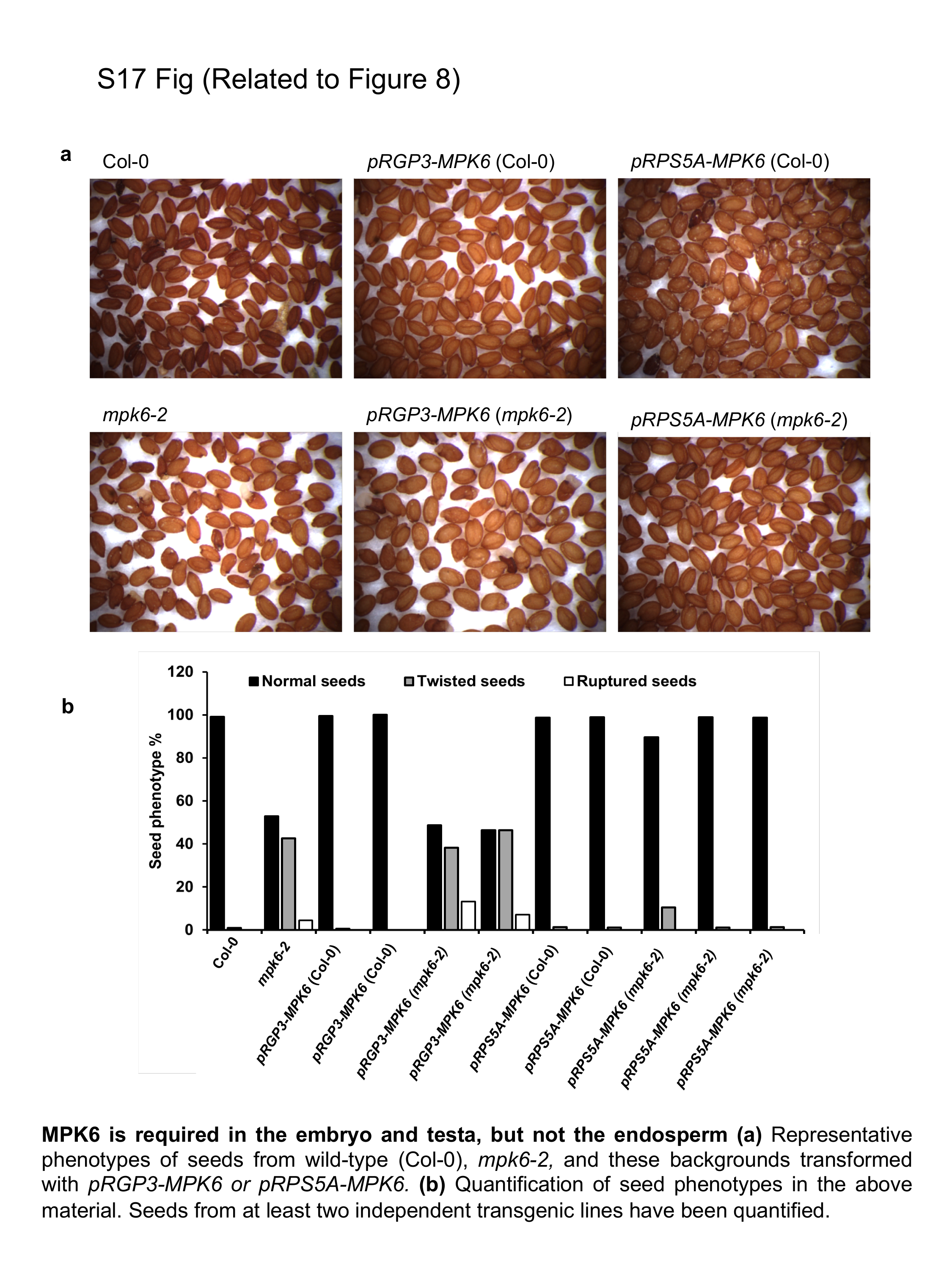

Supplement: S17 Fig — (a) Representative phenotypes of seeds from wild-type (Col-0), mpk6-2, and these backgrounds transformed with pRGP3-MPK6 or pRPS5A-MPK6. (b) Quantification of seed phenotypes in the above material. Seeds from at least two independent transgenic lines have been quantified. (TIF) [file pgen.1007847.s017.tif]

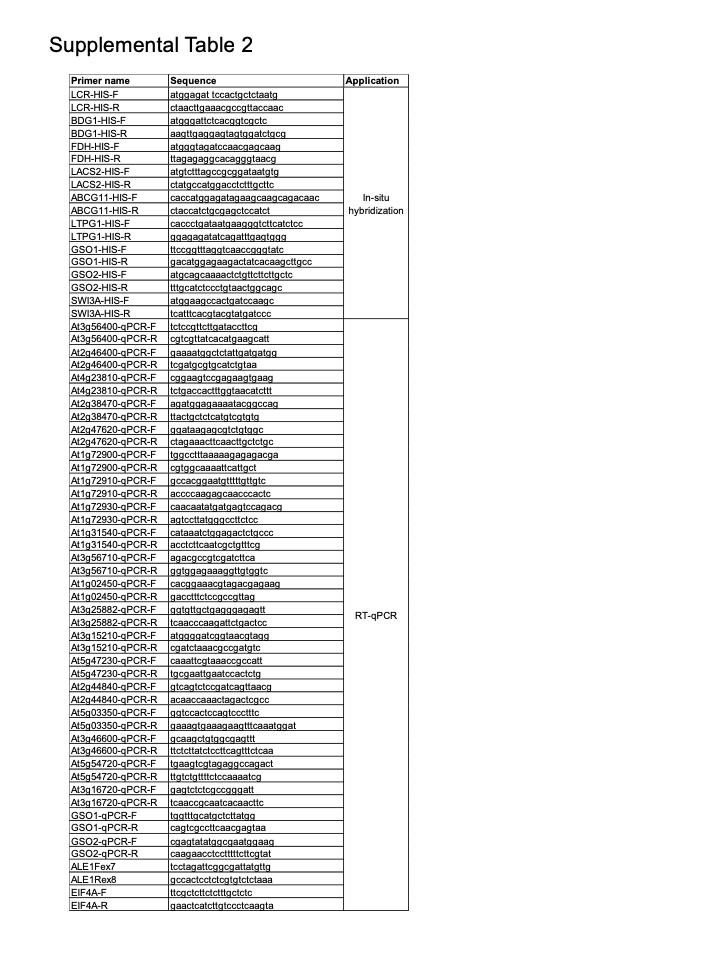

Supplement: S2 Table — (TIFF) [file pgen.1007847.s019.tiff]
